# Molecular Mechanism of Disulfide Bond Healing and Network Repair in Epoxy Vitrimers Revealed by Quantum Chemical and Molecular Dynamics Simulations

**DOI:** 10.3390/polym18070861

**Published:** 2026-03-31

**Authors:** Tomoya Uyama, Naoki Kishimoto, Yutaka Oya, Takahiro Murashima, Jun Koyanagi

**Affiliations:** 1 Department of Materials Science and Technology, Tokyo University of Science, Tokyo 125-8585, Japan; 8224509@ed.tus.ac.jp (T.U.); koyanagi@rs.tus.ac.jp (J.K.); 2 Institute for Excellence in Higher Education, Tohoku University, Sendai 980-8576, Japan; 3 Department of Physics, Tohoku University, Sendai 980-8579, Japan; murasima@tohoku.ac.jp

**Keywords:** molecular dynamics simulation, quantum chemical calculation, vitrimer

## Abstract

We investigate covalent bond healing and mechanical property recovery in a cross-linked epoxy vitrimer containing disulfide bonds by combining quantum chemical calculations and molecular dynamics simulations. Quantum chemical calculations based on the GRRM method are first performed to explore energetically accessible post-scission recombination pathways of sulfur-centered radicals generated by disulfide bond cleavage. The resulting energetic ordering of bonding configurations is incorporated into molecular dynamics simulations through recombination rules derived from the quantum chemical calculations, allowing assessment of network repair and mechanical response. The results indicate that sulfur-centered radicals can undergo post-scission recombination via transient interactions with the aromatic ring prior to reformation of the disulfide bond. Tensile simulations further show that disulfide bonds preferentially break compared with other covalent bonds in the cross-linked network. Incorporation of the recombination pathways identified by the quantum chemical calculations leads to enhanced bond reformation and partial recovery of mechanical properties compared with a model assuming direct sulfur–sulfur recombination only.

## 1. Introduction

Carbon fiber-reinforced plastics (CFRPs) are widely used in structural components for aircraft and other transportation systems owing to their outstanding specific strength and stiffness [1]. However, CFRPs are heterogeneous composite materials consisting of reinforcing fibers and a thermosetting resin matrix, and various types of damage can accumulate during service. It is well known that many of these damage modes originate from the thermosetting resin matrix, where microscopic rupture of covalent cross-links serves as the initial trigger for crack initiation and subsequent growth [2,3]. Once broken, these covalent bonds are essentially irreversible in conventional aerospace-grade thermosets, which poses a major obstacle to extending the service life and reusability of composite structures.

To address this limitation, vitrimers—thermosetting polymers characterized by dynamic covalent networks—have attracted increasing attention in recent years. Vitrimers can rearrange their network topology through bond exchange reactions under elevated temperatures, enabling stress relaxation, reprocessability, and partial recovery of mechanical properties [4]. Numerous molecular dynamics (MD) studies have demonstrated that such dynamic bond exchange plays an important role in enhancing the thermomechanical performance of vitrimer systems [5,6,7,8,9].

However, bond exchange reactions alone do not necessarily guarantee the repair of broken covalent bonds. Bond exchange reorganizes network connectivity, but once a covalent bond has been cleaved, the original bonding pair is not restored. From the viewpoint of damage mechanics, this distinction is crucial: microscopic damage in thermosetting resins is initiated by bond scission, and therefore the recovery of mechanical properties ultimately requires not only topological rearrangement but also reformation of the broken covalent bonds themselves. Despite its importance, the molecular-level processes by which cleaved covalent bonds may heal in vitrimer networks have not been fully clarified.

**Figure 1:**
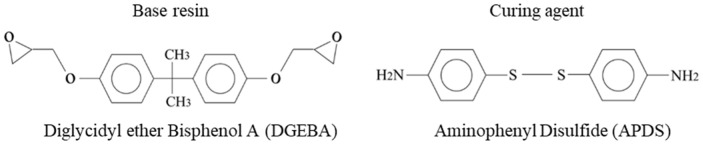
Chemical structures of DGEBA and APDS.

In this study, we focus on covalent bond healing in epoxy vitrimers, rather than on conventional bond exchange reactions. As a representative system, we consider an epoxy vitrimer composed of diglycidyl ether of bisphenol A (DGEBA) cured with 4-aminophenyl disulfide (APDS) [10], the chemical structures of which are shown in Figure 1. APDS contains disulfide (S–S) bonds, which are well known to participate in dynamic exchange reactions. Here, instead of exchange processes, we investigate how a disulfide bond that has been cleaved into two sulfur-centered radicals can subsequently recombine to regenerate the original bond and thereby repair the cross-linked network structure. In the following, the term “APDS fragment” refers to a molecular species generated by cleavage of the disulfide bond in an APDS molecule, in which the sulfur atom exists as a sulfur-centered radical.

Sulfur-centered radicals generated by disulfide bond cleavage exhibit a more three-dimensionally delocalized and polarizable electron distribution than many carbon-centered radicals typically generated upon covalent bond scission in conventional cross-linked epoxy resins [11,12,13]. In such systems, homolytic cleavage of carbon–carbon bonds often produces highly reactive carbon-centered radicals that readily undergo side reactions such as hydrogen abstraction and therefore only infrequently recombine with their original bonding partners [14]. This enhanced delocalization and polarizability impart greater intrinsic stability and longer lifetimes to sulfur-centered radicals, increasing the likelihood of recombination between the two sulfur species generated upon disulfide bond cleavage [11,12,13,15]. In a cross-linked network, such reversible recombination events can provide direct molecular-level pathways for healing broken covalent bonds and restoring network integrity from mechanically damaged, non-equilibrium states, rather than relying solely on equilibration near thermodynamic equilibrium [16,17,18,19].

To elucidate this mechanism, we first perform quantum chemical calculations to explore energetically accessible recombination pathways and intermediate bonding configurations associated with sulfur-centered radicals derived from APDS fragments after disulfide bond scission. The present analysis does not aim to establish rigorous kinetic activation barriers, but rather to identify molecular-level bonding processes that are energetically accessible following bond rupture in a cross-linked network. We then incorporate the bonding processes identified by quantum chemical calculations into molecular dynamics simulations of a cross-linked epoxy vitrimer network. This combined quantum chemical–molecular dynamics approach allows us to directly evaluate how molecular-scale bond healing contributes to network repair and mechanical property recovery in the condensed phase.

By explicitly linking covalent bond scission, post-scission recombination processes, and macroscopic mechanical response, this study provides a molecular-level framework for understanding damage repair in epoxy vitrimer networks. The insights obtained here are expected to contribute to the rational design of damage-tolerant and long-life thermosetting polymer materials that go beyond conventional bond exchange-based vitrimer concepts.

The remainder of this paper is organized as follows. Section 2 describes the quantum chemical methods employed in this study and presents the corresponding results and discussion on the self-healing reaction pathways of molecular species generated by disulfide bond cleavage. Section 3 introduces the molecular dynamics simulation methodology and discusses the deformation behavior, bond breakage, and mechanical property recovery of the cross-linked network. Finally, the main findings of this study are summarized in the Conclusion.

## 2. Exploration of Post-Scission Recombination Pathways by Quantum Chemical Calculations

### 2.1. Computational Methods

Quantum chemical calculations are carried out to investigate local chemical processes related to bond exchange and post-scission bond healing in an epoxy vitrimer system. The target molecular species are derived from 4-aminophenyl disulfide (APDS), which is used as the curing agent in the epoxy vitrimer studied in this work.

**Figure 2:**
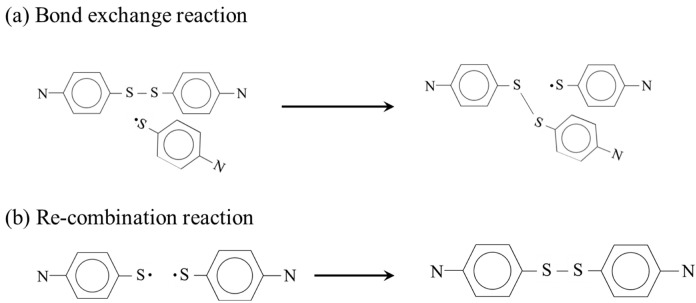
Chemical reactions targeted by our quantum chemical calculations.

In the present study, the term APDS fragment refers to a molecular species generated by cleavage of the disulfide (S–S) bond in an APDS molecule, resulting in a sulfur-centered radical. Based on these molecular species, two types of chemical processes are examined as shown in Figure 2. The first is (i) a disulfide bond exchange process between an intact APDS molecule and an APDS fragment (Figure 2a). The second is (ii) post-scission recombination processes between two APDS fragments (Figure 2b), which are investigated as possible molecular processes responsible for covalent bond healing. The reaction pathway calculations are carried out using the Global Reaction Route Mapping (GRRM) algorithm [20,21], which controls a quantum chemical calculation engine, Gaussian16 [22].

For case (i), the disulfide bond exchange process is investigated using a two-molecule system consisting of one intact APDS molecule and one APDS fragment. Two equilibrium structures that differ only in the connectivity of the disulfide bond are defined as the initial and final states, and reaction pathways connecting these structures are explored using the GRRM method.

For case (ii), post-scission recombination processes between two APDS fragments are investigated starting from configurations corresponding to the fragmented state after disulfide bond cleavage. To avoid assuming a specific recombination mechanism in advance, the two APDS fragments are computationally brought into proximity from multiple random relative orientations, where they interact only weakly at the initial stage. Based on these initial configurations, GRRM calculations are performed to explore possible connectivity changes and transitions between different bonding states that may occur between the two fragments following bond scission.

All quantum chemical calculations are performed using density functional theory at the B3LYP/6-31+G(d) level [23,24,25]. Relative energies discussed in this study are evaluated with respect to reference states appropriate to each process. In particular, for post-scission recombination processes, relative energies are evaluated using reference states appropriate to the purpose of each analysis. For clarity in presentation, the energy of the reformed disulfide-bonded structure is used as the reference (zero energy).

### 2.2. Results and Discussion

**Figure 3:**
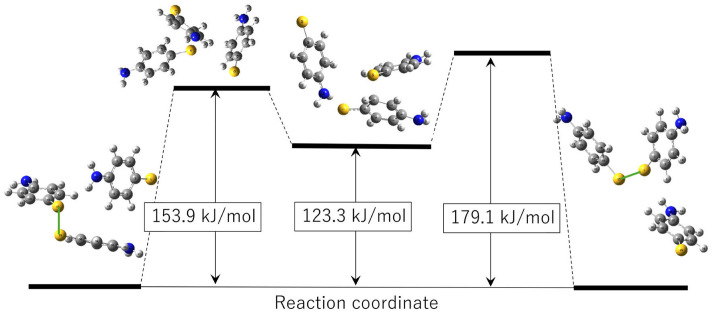
Reaction coordinate of the bond exchange reaction obtained from quantum chemical calculations. Representative molecular conformations along the reaction pathway are shown in ball-and-stick representation, including intermediate states. Carbon, hydrogen, sulfur, and nitrogen atoms are depicted in gray, white, yellow, and blue, respectively, and the disulfide bond is highlighted by green sticks.

First, the results of the disulfide bond exchange process between an intact APDS molecule and an APDS fragment are presented. The reaction coordinate profile obtained from the GRRM calculations is shown in Figure 3, together with representative molecular structures along the pathway. In this process, the reactant and product states consist of the same molecular components and differ only in the connectivity of the disulfide bond. As a result, their energies are essentially identical.

Along the pathway connecting the reactant and product states, one intermediate state and two transition states are identified. These intermediate and transition states involve temporary aggregation of three APDS-derived units, indicating that the bond exchange process proceeds through a multi-molecular configuration rather than a simple two-body interaction. The energetic barriers separating the reactant (or product) state from the intermediate state are on the order of 150–180 kJ/mol.

These energetic features are comparable to previously reported values for disulfide bond cleavage, which are typically around 190 kJ/mol [26]. This similarity suggests that the energetic cost of the bond exchange process is largely governed by disulfide bond breaking, partially compensated by stabilization arising from transient molecular aggregation. Because of these relatively high energy barriers, the disulfide bond exchange process is considered to become relevant predominantly under elevated-temperature conditions, consistent with the characteristic thermal behavior of vitrimer systems.

It should also be noted that the reactant and product structures are essentially identical. Therefore, in principle, the activation energies along the forward and reverse directions of the reaction pathway are expected to be symmetric. However, small deviations from symmetry are observed in the present calculations. These deviations are attributed to residual effects of the internal degrees of freedom of the molecules.

**Figure 4:**
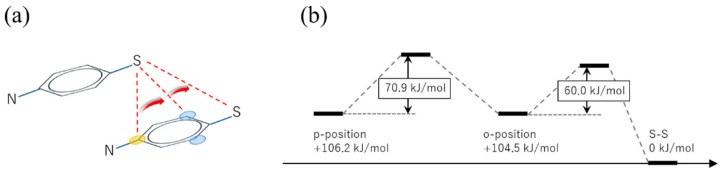
(**a**) Schematic illustration for the reaction pathway from two APDS fragments to an APDS molecule. (**b**) Reaction coordinate of the self-healing reaction.

Next, post-scission recombination processes between two APDS fragments are examined. Figure 4 shows representative bonding configurations explored in the quantum chemical calculations and the corresponding relative energy profile. In contrast to the bond exchange process, this analysis starts from the fragmented state after disulfide bond cleavage and focuses on bonding configurations that may become accessible between two APDS fragments following bond rupture.

Several bonding configurations are identified in which the sulfur-centered radical of one APDS fragment is located near the benzene ring of the other fragment. In particular, configurations associated with the para and ortho carbon positions of the benzene ring are found. When the energy of the reformed disulfide-bonded structure is taken as the reference, these sulfur–carbon-associated configurations lie at higher energies, indicating that they are less stable than the intact APDS molecule.

The relative energies of the recombined bonding configurations provide insight into the possible recombination pathways following disulfide bond cleavage. Here, the energy of the disulfide-bonded state is taken as the reference (0 kJ/mol). When a sulfur-centered radical on an APDS fragment forms a covalent bond with a carbon atom at the ortho position of the aromatic ring on another APDS fragment, the resulting configuration lies approximately 105 kJ/mol above the disulfide bonded state. A similar configuration involving bonding at the para position is slightly higher in energy, at approximately 106 kJ/mol.

In contrast, configurations in which the two APDS fragments are not covalently bonded but remain in close proximity exhibit lower relative energies. Specifically, the non-bonded configuration accessed after cleavage of the para-linked structure is approximately 71 kJ/mol lower in energy than the para-bonded state. Likewise, dissociation of the ortho-linked structure leads to a non-bonded configuration that is approximately 60 kJ/mol lower in energy than the corresponding ortho-bonded state. These energy differences do not represent kinetic activation barriers. Rather, they reflect the relative energetic stability of post-scission bonding and non-bonding configurations accessible after disulfide bond cleavage.

From this viewpoint, the results indicate that the sulfur-centered radical is energetically less stable near the para position than near the ortho position. This energetic ordering suggests that, after bond scission, the radical preferentially localizes toward the ortho region of the benzene ring, which is energetically closer to the reformed disulfide-bonded structure. Such a preference provides a plausible energetic driving force for migration of the radical along the benzene ring prior to reformation of the disulfide bond.

These sulfur–carbon-associated configurations are not interpreted as stable covalent bonds in the conventional sense, but rather as transient or metastable bonding states that may become accessible in mechanically damaged, non-equilibrium environments. Notably, such configurations are observed only in systems composed of two APDS fragments and do not appear when an APDS fragment interacts with an intact APDS molecule.

To further clarify the electronic nature of these sulfur–carbon-associated states, Mulliken bond population analysis was performed for the stable structures obtained from the quantum chemical calculations [22]. Mulliken bond population is derived from the electron density obtained by molecular orbital calculations and provides a measure of electron sharing between two atoms, allowing relative evaluation of bonding interactions.

**Figure 5:**
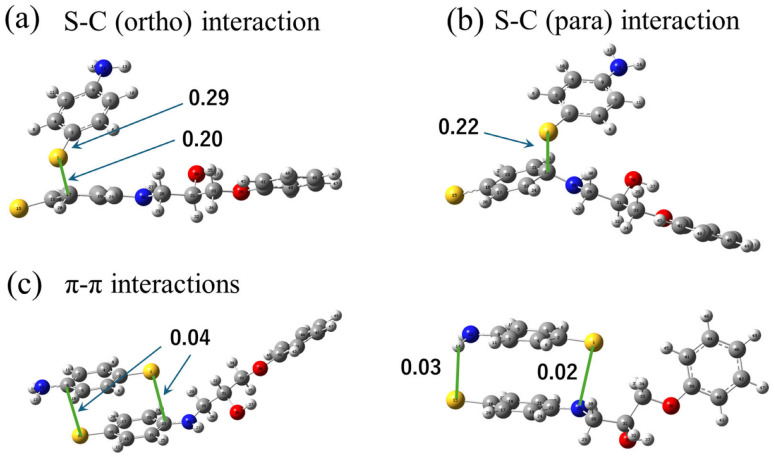
Mulliken bond population analysis of sulfur–carbon-associated configurations obtained from quantum chemical calculations. (**a**) Optimized S–C bonded structure at the ortho position, showing the Mulliken population of the intrinsic S–C σ bond and the newly formed S–C (ortho) interaction. (**b**) Optimized S–C bonded structure at the para position with the corresponding Mulliken population. (**c**) Representative π–π stacking configurations involving sulfur and the aromatic ring, together with the corresponding Mulliken populations.

As shown in Figure 5, the Mulliken population of the intrinsic S–C σ bond within the same APDS molecule is approximately 0.29. In contrast, the newly identified S–C interaction states at the ortho and para positions exhibit values of approximately 0.20–0.22, which are significantly larger than those corresponding to π-stacking–type noncovalent interactions (approximately 0.02–0.04).

Although Mulliken populations depend on the basis set and should not be interpreted as absolute bond orders, comparison within the same theoretical framework indicates that the ortho and para S–C configurations involve appreciable electron sharing. These results support the interpretation that the identified S–C states possess partial covalent character and can be understood as transient bonding configurations along the recombination pathway.

Taken together, these results reveal a clear qualitative difference between the disulfide bond exchange process and the post-scission recombination processes. The bond exchange process involves relatively high energetic barriers and requires elevated temperatures, whereas the post-scission recombination processes involve energetically accessible bonding configurations that become available only after bond rupture. This distinction suggests that post-scission recombination can contribute to covalent bond healing and network repair under conditions where conventional bond exchange is inactive.

## 3. Molecular Dynamics Simulations of Deformation and Self-Healing Behavior

### 3.1. Computational Methods

The molecular dynamics (MD) simulations in this study consist of four consecutive steps: (1) construction of a cross-linked network structure by sequentially introducing chemical reactions between epoxy and amine groups, (2) reproduction of a damaged state by tensile simulations in which covalent bond rupture is allowed, (3) simulation of the damage recovery process by introducing disulfide bond re-formation based on quantum-chemically informed reaction rules, and (4) evaluation of the mechanical properties by applying uniaxial deformation to the structure after damage recovery. The calculation conditions for these processes are described below. All molecular dynamics simulations in this study were carried out using the Large-scale Atomic/Molecular Massively Parallel Simulator (LAMMPS) [27,28]. The chemical reactions introduced in steps (1) and (3), namely the formation of covalent bonds between epoxy and amine groups during network construction and the re-formation of disulfide bonds during the damage recovery process, were implemented using the fix bond/react command in LAMMPS [29,30,31].

#### 3.1.1. Reproducing Cross-Linked Structures

Molecular models of the APDS curing agent and the DGEBA epoxy resin are prepared, and atom types and parameters are assigned to each molecule according to the Polymer Consistent Force Field (PCFF) [32,33]. These molecules are then placed in the simulation cell at an equimolar ratio of functional groups, corresponding to APDS:DGEBA = 1:2, and the total number of atoms is adjusted to be approximately 27,000 atoms. After mixing the molecules, an equilibration run is carried out in the NPT ensemble (P = 1 atm, T = 300 K) to obtain a well-relaxed condensed system in the liquid state, which is used as the initial configuration for the curing simulation.

**Figure 6:**
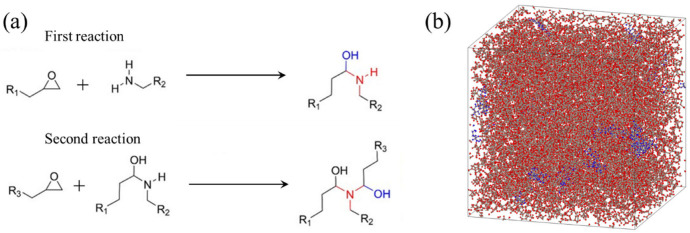
(**a**) Reaction pathway between epoxy and amine groups. (**b**) Snapshot of the cross-linked network structure obtained from the MD simulation, demonstrating the formation of a three-dimensional network in which most molecules belong to a single connected cluster.

During curing, the primary amine groups in APDS are allowed to react with nearby epoxy groups in DGEBA up to two times (Figure 6a), so that three-arm branching nodes are gradually formed. As these branching points spread throughout the simulation cell, they become connected to one another and develop into a highly cross-linked three-dimensional network in which the majority of molecules belong to a single cluster. The curing reaction is introduced using a distance-based reaction rule: when the distance between an epoxy group and an amine group becomes 5 Å or less, a new covalent bond is formed to represent the occurrence of a curing reaction. This distance-based reaction rule is widely used in atomistic simulations of epoxy curing to represent the onset of chemical reactions in a coarse-grained manner [34,35,36].

This reaction–relaxation cycle is repeated under NPT conditions, where short relaxation runs are inserted between successive reaction steps so that excessive local stress does not accumulate during network formation. The degree of cross-linking is defined as the ratio of the number of reacted epoxy groups to the total number of epoxy groups prior to curing, and the final conversion reaches approximately 80%, which is comparable to the curing ratio observed in experiments [37]. Figure 6b shows a snapshot of the system after the curing reaction. This snapshot serves to confirm that a well-developed three-dimensional cross-linked network is formed in the MD model, where most molecules are incorporated into a single connected cluster. Atoms belonging to the same molecular cluster are depicted in the same color, while all covalent bonds are shown in gray. As can be seen, most atoms are colored red, indicating that the majority of molecules are incorporated into a single large cluster. This observation is consistent with the formation of a highly cross-linked three-dimensional network.

To evaluate statistical robustness, three independently prepared cross-linked network structures were generated under identical curing conditions. For each structure, tensile simulations were performed along the x, y, and z directions, resulting in nine independent simulations. The stress–strain curves shown below represent the average over these simulations, and the shaded regions indicate the variability among samples.

#### 3.1.2. Tensile Deformation and Damage Introduction

The calculation conditions for the uniaxial deformation analyses conducted in steps (2) and (4) are as follows. The system is deformed along the tensile direction at an engineering strain rate of 5.0 × 10^8^/s, while the cross-sectional area perpendicular to the tensile direction is kept fixed during deformation. This boundary condition is intended to mimic the deformation state of the resin phase in fiber-reinforced composites, where the lateral deformation of the resin is constrained by the reinforcing fibers [38]. The temperature is maintained at T = 300 K throughout the simulations.

Covalent bond rupture during tensile loading is introduced using a distance-based criterion. Candidate bonds for rupture include all covalent bonds except carbon–carbon bonds forming the aromatic ring backbone and bonds involving hydrogen atoms [39,40]. For each bond type, a reference maximum covalent bond length was defined based on quantum chemical calculations, corresponding to the largest bond distance classified as a covalent bond by the GRRM algorithm [21]. The rupture threshold is then defined as 0.64 times this reference bond length. When a bond length exceeds the threshold value during deformation, the corresponding bond is removed from the system.

It should be noted that bond-breaking distances obtained from quantum chemical calculations correspond to idealized molecular configurations in the absence of topological constraints. In a highly cross-linked polymer network, however, structural frustration arising from network connectivity can promote bond rupture at shorter bond lengths than those predicted under ideal conditions. To account for this effect, a reduced rupture threshold is adopted in the present simulations. The value of the threshold is calibrated through preliminary simulations such that the peak stress in the stress–strain response appears at a strain of approximately 0.1, which is comparable in magnitude to experimentally observed failure strains of epoxy resins. Although bulk mechanical properties emerge from larger-scale structural features, atomistic simulations provide essential insight into how bond rupture and recombination govern stress transfer at the molecular level.

#### 3.1.3. Damage Recovery Simulations Through Recombination Reactions

The calculation conditions for the damage recovery simulations conducted in step (3) are described below. During the uniaxial tensile simulations in step (2), disulfide bonds in the cross-linked network are allowed to rupture, resulting in the generation of a large number of APDS fragments within the system. The subsequent damage recovery process of the network is modeled by reproducing, within the MD framework, recombination processes between APDS fragments that are suggested by the quantum chemical calculations.

First, the damaged network structure obtained after the tensile simulation is equilibrated by performing an NPT simulation (P = 1 atm, T = 300 K) for 5 ns. After this relaxation, recombination reactions associated with the re-formation of disulfide bonds are introduced sequentially using a distance-based criterion. Specifically, a new covalent bond is created when the distance between two atoms reaches the maximum covalent bond distance identified from the quantum chemical calculations.

The recombination reactions introduced in the MD simulations correspond to the following five types of bonding processes suggested by the quantum chemical analysis: (i) direct recombination between the sulfur atoms of two APDS fragments, (ii) bond formation between a sulfur atom of an APDS fragment and a carbon atom at the ortho position of the benzene ring, (iii) bond formation between a sulfur atom of an APDS fragment and a carbon atom at the para position of the benzene ring, (iv) transition from a sulfur atom bonded at the ortho position to a disulfide bond, and (v) transition from a sulfur atom initially bonded at the para position to a bond with a carbon atom at the ortho position.

In the present MD model, kinetic activation energies are not explicitly considered. Instead, the recombination processes are implemented based on the energetic ordering and relative stability of bonding configurations identified in the quantum chemical calculations. Considering the relative energetic accessibility of these configurations, the recombination process is expected to proceed in a predominantly directional manner on experimentally relevant time scales. Specifically, the bonding state of the sulfur-centered radical tends to shift from the para position to the ortho position on the benzene ring, and subsequently transitions into a sulfur–sulfur bond. This directional nature of the recombination process is incorporated into the MD simulations to represent damage recovery in the cross-linked network.

### 3.2. Results and Discussion

**Figure 7:**
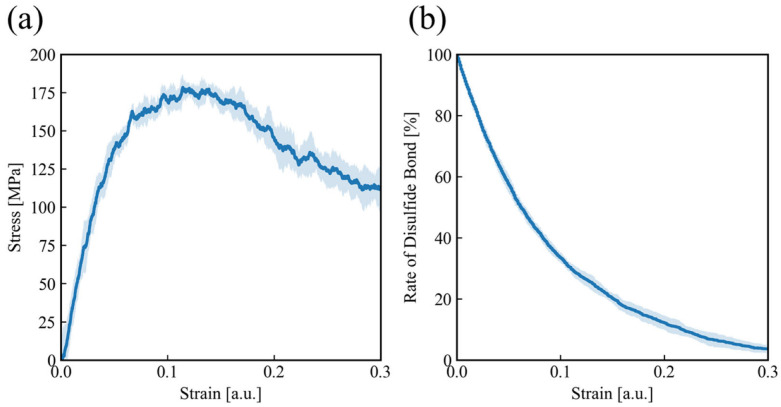
(**a**) Stress–strain curve of the cross-linked epoxy vitrimer network obtained from uniaxial tensile deformation with explicit covalent bond breakage. (**b**) Fraction of disulfide bonds remaining in the system as a function of applied tensile strain.

First, uniaxial tensile simulations are performed for the cross-linked network composed of APDS and DGEBA, explicitly allowing covalent bond breakage. The resulting stress–strain curve is shown in Figure 7a. From this curve, Young’s modulus is estimated to be approximately 5.0 GPa, and the tensile strength is about 180 MPa. These values are slightly higher than those reported experimentally for epoxy resin specimens; however, this difference is reasonable because the deformation is applied under a condition in which the cross-sectional area perpendicular to the tensile direction is fixed. The strain at the maximum stress is approximately 0.1. Although this value is larger than typical experimental failure strains of epoxy resins, which are often on the order of a few percent, it is of the same order of magnitude and is regarded as physically reasonable within the simplified bond-breaking framework employed in the present MD simulations. Although the rupture threshold was calibrated to reproduce a physically reasonable failure strain, additional simulations were conducted to confirm that moderate variations in the threshold do not alter the qualitative deformation behavior. When the correction factor varied from 0.625 to 0.65 of the reference bond length in increments of 0.05, the strain at peak stress and the selective rupture of disulfide bonds remained unchanged. The tensile strength varied moderately (approximately 170–220 MPa), while the preferential breakage of S–S bonds and the subsequent healing behavior were consistently observed. These results indicate that the mechanistic conclusions are robust with respect to the rupture criterion within this range.

Figure 7b shows the relationship between strain and the fraction of disulfide bonds remaining in the system. As the strain increases, the number of broken bonds increases monotonically. An important observation is that all broken bonds are disulfide bonds, while no other covalent bonds in the network fracture under the present loading conditions.

This behavior is explained by the mechanical characteristics of the disulfide bonds. Compared with other covalent bonds, disulfide bonds exhibit a smaller critical strain for bond breakage when measured relative to their equilibrium bond length. In addition, the spring constant of the bond potential for disulfide bonds is smaller than that of other covalent bonds. As a result, disulfide bonds are more susceptible to rupture under tensile deformation.

When disulfide bonds break, the load supported by the remaining covalent bonds in the network is partially released. This load redistribution prevents other covalent bonds from breaking, leading to selective fracture of disulfide bonds only. Such a mechanism, in which mechanically weaker bonds are uniformly distributed in the cross-linked network and preferentially broken, suppresses the simultaneous rupture of many covalent bonds caused by stress concentration, which has been observed in the previous experimental study [41]. Consequently, this selective bond breakage contributes to an increase in the fracture strain of the system.

Next, the self-healing behavior of the damaged cross-linked network is investigated. The cross-linked structure is first subjected to uniaxial tensile deformation up to a strain of 200% to induce sufficient cleavage of disulfide bonds. After deformation, the system is relaxed under NPT conditions to obtain an equilibrated damaged structure. Subsequently, the self-healing reactions between APDS fragments identified by quantum chemical calculations are introduced to repair the cross-linked network.

**Figure 8:**
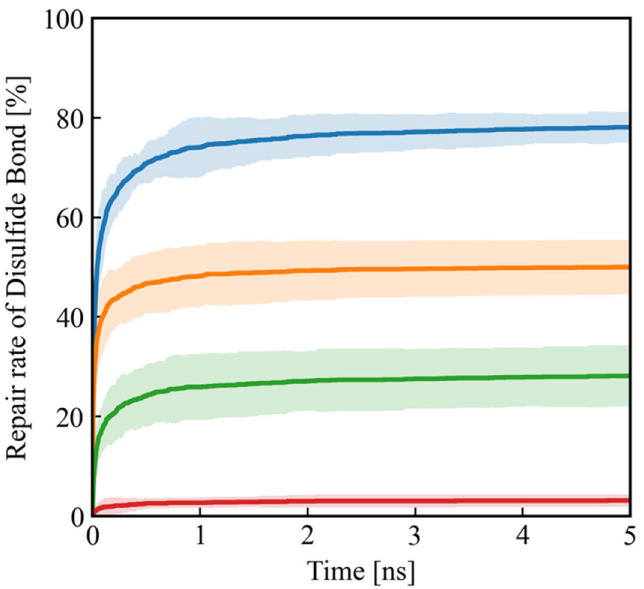
Time evolution of the repair rate of disulfide bonds during the damage recovery simulation. The blue curve represents the total fraction of disulfide bonds reformed in the system. The orange, green, and red curves indicate contributions from direct sulfur–sulfur recombination and recombination pathways involving transient bonding at the ortho and para positions of the benzene ring, respectively.

Figure 8 shows the time evolution of the number of recovered bonds during the relaxation process. The formation of covalent bonds and transitions between bonding states nearly saturate within approximately 5 ns. As a result, about 78% of the broken disulfide bonds are reformed by the end of the simulation. The remaining approximately 22% of sulfur radicals originating from APDS fragments do not recombine, which is attributed to geometric constraints in the cross-linked network that prevent these radicals from finding their corresponding partners. This result indicates that the damaged cross-linked structure does not fully return to its original topology even after the self-healing reactions occur.

A closer examination of the self-healing pathways reveals that approximately 64% of the reformed disulfide bonds originate from direct recombination between sulfur-centered radicals, while the remaining 36% are formed via recombination pathways involving transient bonding at the ortho position of the benzene ring. Although direct sulfur–sulfur recombination constitutes the majority of healing events, the ortho-mediated pathway accounts for a substantial fraction of bond reformation. This result demonstrates that the recombination pathway identified by the quantum chemical calculations contributes non-negligibly to the repair of the cross-linked network.

**Figure 9:**
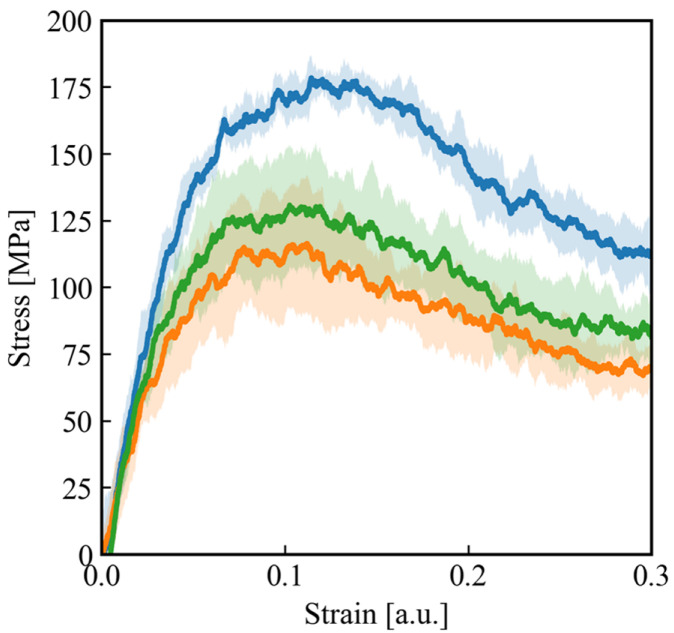
Stress–strain curves obtained from uniaxial tensile deformation of three different network states: the undamaged initial cross-linked structure (blue), the damaged structure equilibrated without self-healing reactions (orange), and the damaged structure after applying the self-healing reactions (green).

Finally, a comparison of the stress–strain curves for three different structures is shown in Figure 9. The blue curve corresponds to the deformation of the undamaged initial cross-linked structure and is identical to that shown in Figure 7a. The orange curve represents the stress–strain response of the damaged cross-linked structure equilibrated without introducing any self-healing reactions, while the green curve shows the stress–strain behavior of the damaged cross-linked structure after the self-healing reactions are applied. Both Young’s modulus and the tensile strength decrease in the order of the undamaged structure, the self-healed structure, and the damaged structure without healing. These results indicate that the self-healing reactions incorporated into the MD model lead to a partial recovery of mechanical properties compared with the damaged structure, demonstrating the contribution of post-scission recombination processes to network repair.

It should be emphasized that the present molecular dynamics simulations are not intended to provide quantitative prediction of bulk mechanical properties. Macroscopic mechanical behavior emerges from structural heterogeneities and length scales beyond those directly accessible in atomistic simulations. Instead, the objective of this study is to clarify how specific bond-level events—namely selective disulfide bond scission and QC-informed recombination pathways—modify network connectivity and influence relative changes in mechanical response under controlled simulation conditions. Therefore, the mechanical property variations discussed here should be interpreted as mechanistic indicators of network repair at the molecular scale rather than absolute predictions of macroscopic material performance.

## 4. Conclusions

In this study, we investigated covalent bond repair in epoxy vitrimers with disulfide linkages, focusing on post-scission recombination processes rather than conventional disulfide bond exchange reactions that have been extensively discussed in previous studies. Quantum chemical calculations were employed to explore energetically accessible recombination pathways of sulfur-centered radicals generated by disulfide bond cleavage, without assuming explicit reaction kinetics. Based on the identified energetic ordering of bonding configurations, molecular dynamics simulations incorporating QM-informed rule-based recombination processes were performed to analyze deformation behavior, bond scission, and subsequent damage recovery in a cross-linked network.

By integrating quantum chemical calculations with molecular dynamics simulations, the present approach enables direct evaluation of covalent bond repair and its influence on mechanical response in condensed polymer networks, which cannot be captured by quantum chemical calculations on isolated molecular systems alone. The main findings of this study can be summarized as follows.

(1) An energetically accessible post-scission recombination pathway between APDS fragments is identified, in which a sulfur-centered radical transiently interacts with carbon atoms at the para and ortho positions of the benzene ring in the counterpart fragment before reforming the disulfide bond. This pathway represents an alternative bond-healing mechanism distinct from conventional disulfide bond exchange reactions.
(2) Deformation analyses reveal that disulfide bonds preferentially break compared with other covalent bonds in the cross-linked network. Such selective bond scission suppresses simultaneous rupture of multiple covalent bonds, a behavior previously observed in conventional epoxy systems cured with amine-based hardeners, and is therefore considered to contribute to enhanced damage tolerance at the molecular scale.
(3) Incorporation of the QM-informed recombination pathways into molecular dynamics simulations leads to partial restoration of the cross-linked network structure and corresponding recovery of mechanical properties compared with the damaged state, highlighting the contribution of post-scission recombination processes to network repair.

Overall, this study provides a molecular-level framework for understanding covalent bond repair in epoxy vitrimer networks under mechanically damaged, non-equilibrium conditions. The insights obtained here are expected to contribute to the rational design of damage-tolerant and long-life thermosetting materials that extend beyond conventional bond exchange-based vitrimer concepts.

## Data Availability

Data is contained within the article.
